# Bone-strengthening pill (BSP) promotes bone cell and chondrocyte repair, and the clinical and experimental study of BSP in the treatment of osteonecrosis of the femoral head

**DOI:** 10.18632/oncotarget.21226

**Published:** 2017-09-23

**Authors:** Zheng Li, Lulin Wang, Jin Wei, Liguo Zhu, Xisheng Weng, Jin Jin, Hong Xiao, Jun Zhang, Heming Wang, Guantong Shi, Lingpeng Pei, Fangde Zou, Wanqiang Zhang, Tianzun Tao, Xin Dong

**Affiliations:** ^1^ Department of Orthopedic Surgery, Peking Union Medical College Hospital, Chinese Academy of Medical Sciences, Peking Union Medical College, Beijing, 100730, China; ^2^ Department of Orthopedics, Beijing Jianxing Traditional Chinese Medicine Hospital, Beijing, 100007, China; ^3^ Department of Dermatology and Plastic Surgery, China Aerospace 731 Hospital, Beijing, 100074, China; ^4^ Wangjing Hospital of China Academy of Chinese Medical Sciences, Beijing, 100102, China; ^5^ Fujian Provincial Institute of Traditional Chinese Medicine, Fuzhou, 350003, China; ^6^ Shuguang Hospital, Shanghai University of Traditional Chinese Medicine, Shanghai, 200021, China; ^7^ Traditional Chinese Medicine, University of MINZU, Beijing, 100081, China; ^8^ Patent Office, Tongliao Municipal Science and Technology Bureau, Tongliao, 028000, China; ^9^ Department of Orthopedics, The 2nd Affiliated Hospital of Harbin Medical University, Harbin, 150001, China; ^10^ Department of Radiology, Beijing Zhongguancun Hospital, Beijing, 100190, China

**Keywords:** osteonecrosis of femoral head, bone and cartilage, bone and joint disease, treatment, bone-strengthening pill

## Abstract

About 1 in 3 people suffer from bone and joint disease, which is a disease of bone and cartilage cells. Osteonecrosis of the femoral head (ONFH) is a typical example of bone and joint disease involving bone cell necrosis. Osteonecrosis of the femoral head leads to the occurrence of premature osteoarthritis of the hip and collapse of the cartilage cells, and there is currently no effective drug treatment available. In order to study the effects of “bone-strengthening pill” (BSP) on the repair of bone and cartilage cells, we investigated the potential effects of the herbal mixture BSP in an animal model of avascular necrosis of the femoral head and in patients. Results showed that 90% of rats injected with prednisone developed ONFH, whereas BSP administration prevented ONFH development in 70% of prednisone-injected rats. We evaluated the constituents of BSP by HPLC fingerprinting. We also evaluated the clinical efficacy of BSP in a double-blind, randomized, controlled trial of 300 patients with ONFH. The response rate was found to be higher in the treatment group than in the control group, with a response rate of 82% in the treatment group. Treatment with BSP also significantly reduced pain, improved hip function, reduced lameness, and improved pathology by X-ray and MRI analysis, compared with patients who did not receive BSP. These results suggest that BSP treatment inhibits and reverses necrosis of the femoral head bone cells and cartilage cells to repair the femoral head, promote the repair of bone and cartilage diseases.

## INTRODUCTION

About 1 of every 3 people suffers from bone and joint disease, which is a disease of bone and cartilage cells. Osteonecrosis is a disabling disease of multiple bone and joints caused by the degeneration of bone tissue including bone marrow cells, adipocytes, and subsequent necrosis.

Osteonecrosis of the femoral head (ONFH) is a typical bone and joint disease involving necrosis. Bone and joint diseases that comprises bacterial disease (osteomyelitis and bone tuberculosis) And sterile disease (osteonecrosis of the femoral head necrosis of, the talus, osteonecrosis of the knee necrosis of the osteoarthritis, of the fracture, and nonunion, of the bone infarction, of fibrous dysplasia, etc.) and poses a serious threat to the health of an individual. ONFH is a debilitating form of bone necrosis caused by the progressive interruption of the blood supply, leading to osteocyte and bone marrow cell death and ultimately femoral head collapse [1–4]. The occurrence of ONFH primarily affects men in their fifties, leading to premature hip osteoarthritis and osteochondral collapse [5–7]. In the United States, ONFH accounts for 5%–18% of total hip arthroplasties performed in recent years [8, 9]. The pathogenesis of ONFH is complex, involving genetic susceptibility (e.g., mutations in the *COL2A1* gene and polymorphisms in alcohol metabolizing enzyme genes and multidrug resistance gene 1), and environmental insults (eg corticosteroids and alcohol consumption) [10–14]. Although conservative approaches are available including extracorporeal shock waves and pulsed electromagnetic fields, and surgical remedies such as osteotomies, core decompression, and vascularized or non-vascularized bone grafting, the treatment outcome of ONFH remains unsatisfactory [1, 15–18]. Eighty percent of patients diagnosed with ONFH will eventually need total hip arthroplasty [19, 20]. Currently, there is no effective method for preventing the onset and progression of ONFH.

The herbal mixture bone-strengthening pill (BSP), also called Jiangusheng Wan, comprises an empirical formula of pberetima, pearl, angelica, and pseudo-ginseng and has been used for the treatment of orthopedic diseases for many years [21]. Nevertheless, preclinical and clinical evidence for its therapeutic efficacy is lacking. To investigate the role of BSP in the repair of bone and cartilage diseases, we evaluated the potential curative effect of this herbal mixture and studied its pharmacological mechanism in an animal model of ONFH and in patients with ONFH.

## RESULTS

### Quality evaluation of BSP

**Figure 1:**
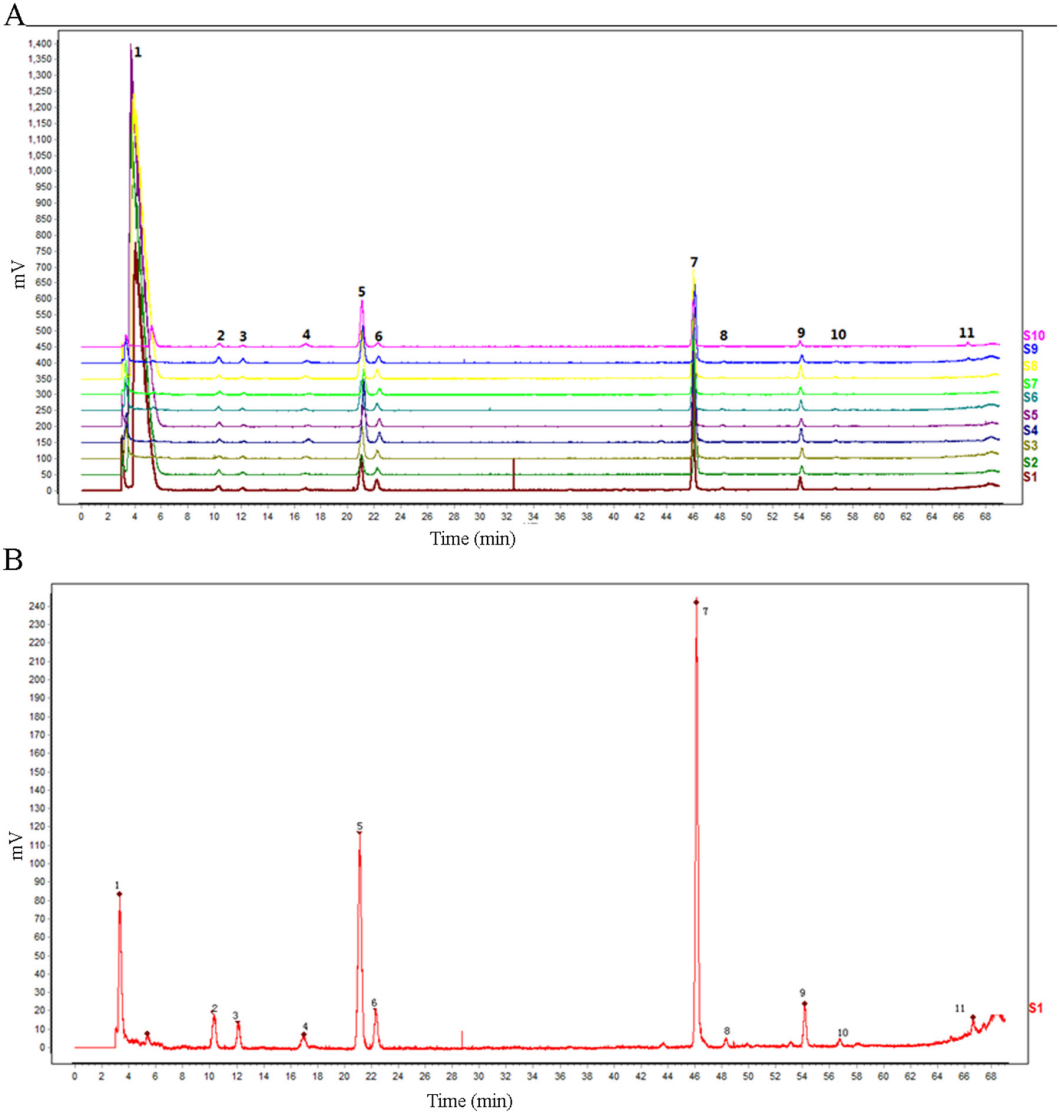
Quality evaluation of the bone-strengthening pill (BSP) The major constituents in 10 samples of BSPs were measured by HPLC fingerprinting.

The major constituents in 10 samples of BSP were measured by HPLC fingerprinting. Eleven peaks were observed (Figure 1A). The first peak was from pberetima and pearl; the peaks 2, 3, 8, and 11were from angelica, and the peaks 4, 5, 6, 7, 9, and 10 were from pseudo-ginseng. Moreover, these peaks were stable even when the pills had been stored for 60 months (Figure 1B).

**Figure 2:**
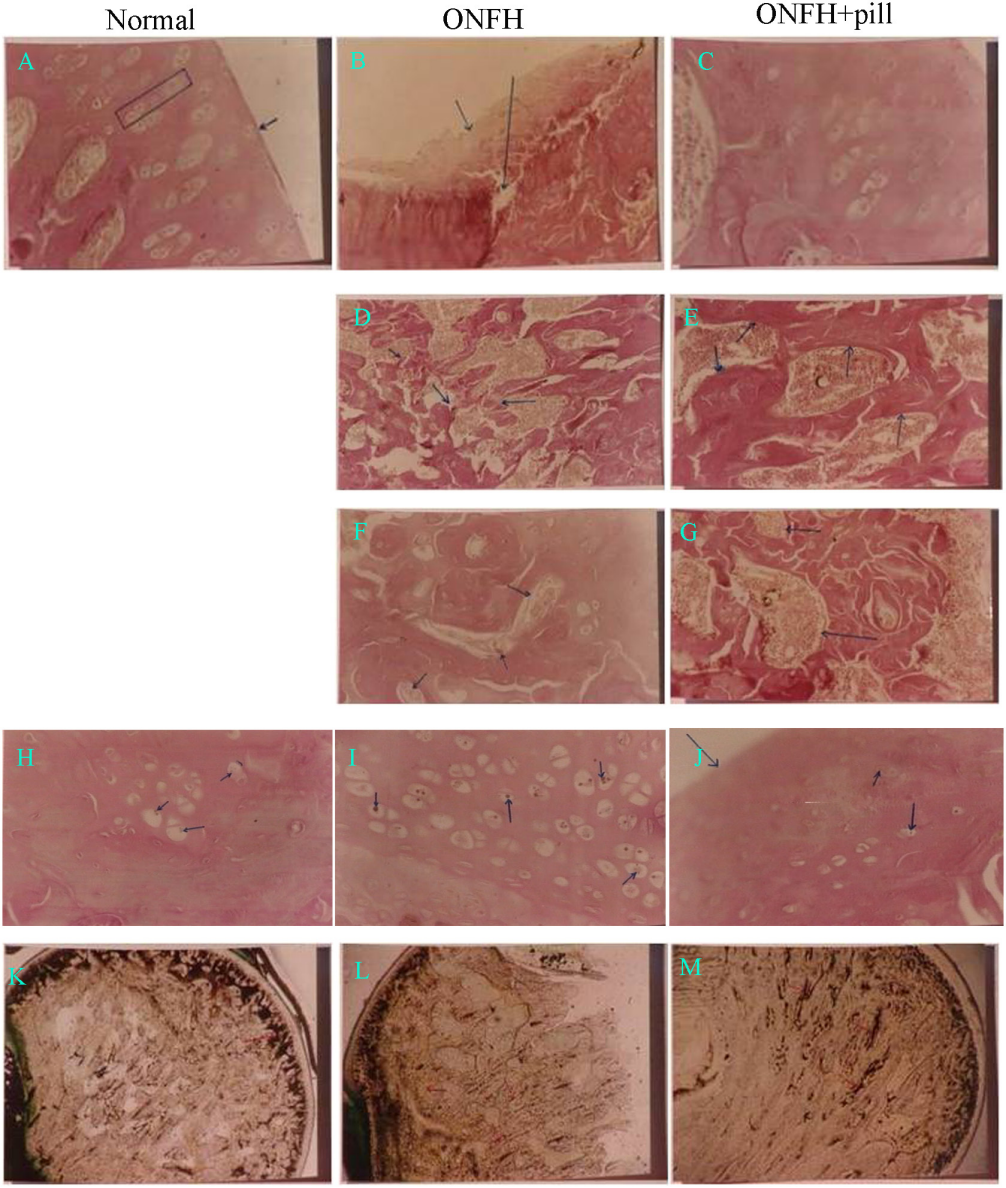
The bone-strengthening pill (BSP) reduces osteonecrotic changes in osteonecrosis of the femoral head (ONFH) in rats Histological observations of the femoral head are shown. **(A)** The surface of the femoral head is smooth and the cartilage cells show columnar growth pattern. **(B)** The surface of the femoral head is necrotic and shredded. Collapse and defectsare observed. **(C)** The surface of the femoral head is smooth and the cartilage cells show columnar growth pattern. **(D)** Curvature, disturbance, and rupture of bone trabecula are observed. **(E)** There is abundant distribution of bone trabecula, which has a normal structure. **(F)** Focal necrosis of marrow cells. **(G)** No necrosis of marrow cells is observed. **(H)** A few fat particles adhering to the surface of the cartilage cells were observed. **(I)** A large amount of fat particles are observed inside the cartilage cells. **(J)** A few fat particles adhering to the surface of the cartilage cells were observed. **(K)** Abundant capillaries with clear marginsare observed to form a capillary network and arcuate cup. **(L)** The capillaries were sparse inside the femoral head and the arcuate cupdisappeared. **(M)** There was a significant increase in capillaries inside the femoral head with clear margins, with recovery of the arcuate cup.

The changes of necrosis of femoral head decreased the BSP of rats Steroids induced necrosis of the femoral head in rats. Ten rats showed necrosis of the femoral head, and 9 showed damage or defects on the surface of the femoral head (Table 1). After treatment with BSP, 70% of the treated rats recovered (Figure 2A–2C). After administration of prednisone, disturbance, curvature and rupture of bone trabecula and focal necrosis of the marrow cells were observed. Additionally, prednisone reduced the bone trabecula volume and width, bone cortex width, and osteoid width of the femoral head but significantly increased the percentage of empty lacunae; all these effects were attenuated by BSP (Table 1B, Figure 2D–2G). Steroids have been shown to induce fat accumulation during ONFH. In our experiments, prednisone significantly increased the number of cells with fat particles, and this number was reduced by BSP treatment (Table 1C, Figure 2H–2J). Prednisoneinduced reduction of the capillary vessels of the femoral head and the disappearance of the arcuate cup of capillary vessels; the effects were also abolished by BSP (Figure 2K–2M). We next assessed serological markers related to bone turnover. Prednisone increased the serum levels of tartrate-resistant acid phosphatase (TRAP; a biomarker of bone resorption) but reduced ALP levels (a biomarker of active bone formation). These serological changes were reversed after treatment with BSP (Table 1D).

**Table 1A:** BSP reduces osteonecrotic changes in ONFH rats

Group	N	Type	Samples	N%
Control	10	A	4	40
		B	5	50
		C	1	10
BSP	10	A	0	0
		B	3	30
		C	7	70

A: Break or defect of femoral head surface; B: Only a change of bone cells, cartilage cells, and myeloid cells, no change of femoral head surface; C: Normal.

**Table 1B:** The effect of BSP on the bone

Group	N	TBV%	MTPT	MAR	MOSW
Normal	10	61.80±3.38^**^	108.08±6.17^**^	1.26±0.15^**^	1.63±0.11^**^
Prednisone	10	55.03±5.14	93.53±12.28	0.66±0.11	0.83±0.05
BSP	10	63.73±6.44^**^	108.20±6.56^**^	1.06±0.17^**^	1.44±0.29^**^

Compared to normal group: ^*^P<0.01; compared to prednisone group: ^#^*P*<0.01. Femoral head (TBV), bone trabecula width of femoral head (MTPT), Bone mineralization rate (MAR) and osteoid width (MOSW).

**Table 1C:** The effect of BSP on the number of cells with fat particles

Group	N	Fat deposition rate %	*P*
Normal	10	4.74±3.11	<0.01
Prednisone	10	16.64±7.42	-
BSP	10	4.63±2.22	<0.01

**Table 1D:** The effect of BSP on blood biochemical indices in the rat

Group	n	Result	
		TRAP(B-L)	ALP(U)
Normal	10	2.08±0.24^▲^	5.13±0.96
Prednisone	10	2.40±0.33	4.57±1.44
BSP	10	1.47±0.32^*^	6.55±1.34^*^

^▲^Compared to normal group: *P*<0.05; ^*^compared to prednisone group: *P*<0.01.

### BSP administration reduces osteonecrotic changes in ONFH patients

**Figure 3:**
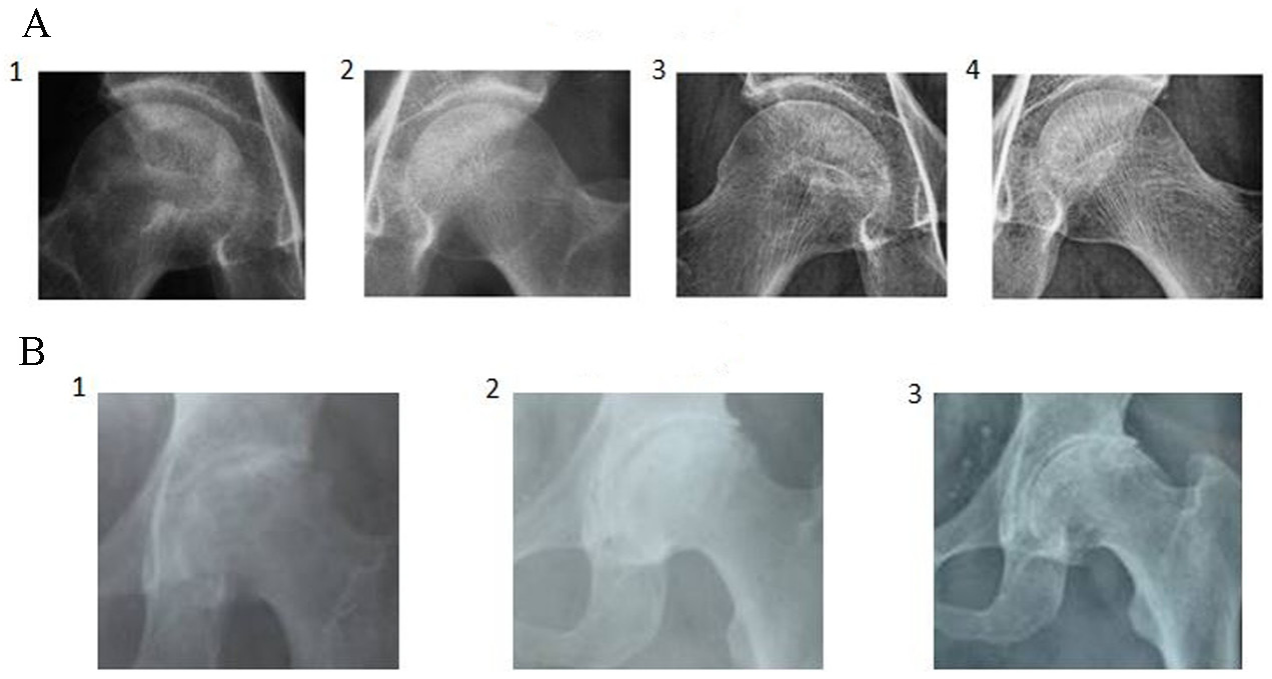
Representative case of osteonecrosis of the femoral head (ONFH) by X-ray analysis **(A)** 52-year-old male patient, alcoholic. 1 and 2: The density of the femoral head was not uniform and bone trabeculae disappeared, especially on the right side. 3 and 4: After treatment with the bone-strengthening pill (BSP), the pathological radiographic findings disappeared and the patient resumed work. **(B)** 58-year-old male patient, no clear etiology was determined. 1: The outline of the femoral head was disrupted with the disappearance of the joint space and loss of bone trabeculae. 2: After treatment with BSP, the surfaces of the femoral head became smooth with joint space restored and clear margins of bone trabeculae. The patient started light work after treatment. 3: A 16-year follow-up showed a smooth surface of femoral head, clear joint space, and bone trabeculae.

**Figure 4:**
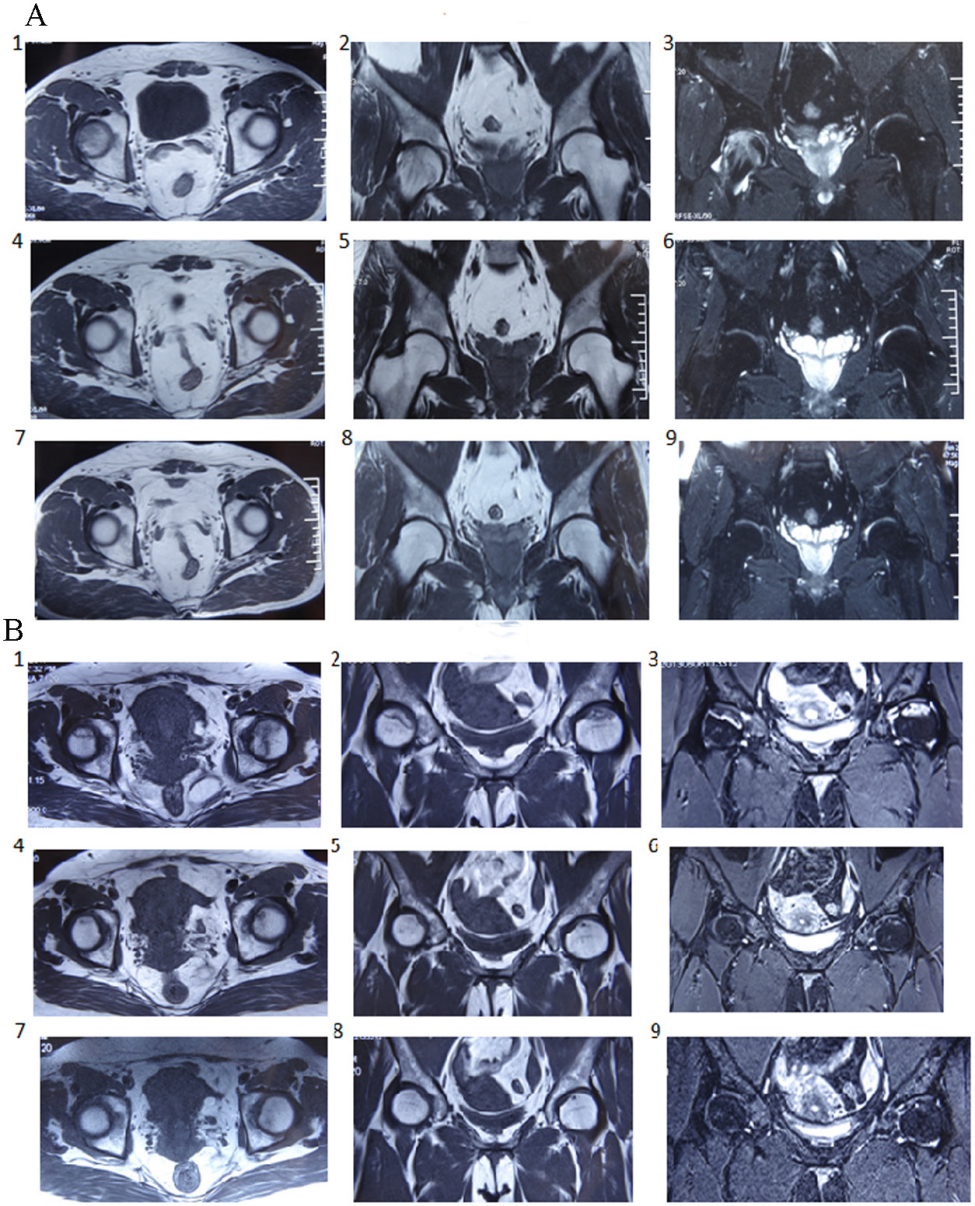
Representative case of osteonecrosis of the femoral head (ONFH) evaluated by magnetic resonance imaging (MRI) **(A)** 54-year-old male patient, alcoholic (250 ml of alcohol per day). 1 - 3: There were abnormal signals on the right side of osteochondritis of the femoral head and an extensive area of abnormal signals under the perichondrium. 4 - 6: After treatment with the bone-strengthening pill (BSP) for 6 months, the abnormal signals disappeared and the patient resumed normal work. 7 - 9: The MRI image had no abnormalities at 1-year follow-up. **(B)** 49-year-old female patient, steroid administration 5 years ago and ONFH for 2 years. 1 - 3: MRI showed slight collapse of the bilateral femoral head. There were large and irregular long T1 and long T2 signals on the anterior superior segment of the bilateral femoral head. STIR revealed that the necrotic lesions exhibited a ladder-like, high, mixed, uneven abnormal signal, disappearance of cartilage signal especially on the left side. 4 - 6: The abnormal signals decreased and the patient could undertake light work after treatment for 6 months. 7 - 9: At 1-year follow up after treatment, the MRI was normal and patient could work as usual.

We evaluated the efficacy of BSP in patients with avascular necrosis of the femoral head using a double-blind, randomized controlled trial. A total of 300 ONFH patients were included in this study (Table 2A). Of these, 100 were salvia tablets were used as the control pills. The effective rate of BSP was 82%, which was significantly higher than that of the control pills (Table 2B). The rate of improvement in pain relief, hip joint function, and claudication was significantly better in the BSP group than in the control group (Table 2C–2F). Sex, age, or etiological subtypes had no effect on the response to BSP (Table 2G–2I). Representative cases are shown in Figure 3A and 3B. Moreover, BSP could significantly ameliorate ONFH as evaluated by X-ray analysis (Table 2J). The results from routine blood, urine, heart, liver, and kidney tests showed that BSP was safe, and BSP significantly ameliorated ONFH as evaluated by MRI analysis (Table 2K, Figure 4A and 4B).

**Table 2A:** Clinical pathologic characteristics of patients

Parameter		BSP	Control	P
Gender	Male	62	65	>0.05
	Female	38	35	
Age	≤40	33	29	>0.05
	>41	67	71	
Etiology	Trauma	31	31	>0.05
	Other	69	69	

**Table 2B:** Curative effect in different groups

Group	N	Excellent	Effective	Invalid	R
BSP	100	35	47	18	0.6644
control	100	0	25	75	0.3356

**Table 2C:** Curative effect of BSP

Group	N	Excellent	Effective	Invalid	N%
BSP	200	62	103	35	82.5

**Table 2D:** Pain improvement of different groups

Group	N	Pre-treatment	Post-treatment
BSP	100	3.20±0.81	1.17±1.05
Control	100	3.34±0.79	2.27±1.00

**Table 2E:** Functional improvement of different groups

Group	N	Pre-treatment	Post-treatment
BSP	100	3.26±0.92	2.00±1.06
Control	100	3.31±0.83	2.89±0.98

**Table 2F:** Claudication improvement of different groups

Group	N	Pre-treatment	Post-treatment
BSP	100	3.23±0.75	1.78±1.06
Control	100	3.14±0.76	2.74±0.97

**Table 2G:** The influence of gender on efficacy in the BSP group

Group	N	Excellence	Effective	Invalid	P
Male	118	37	61	20	0.4569
Female	82	25	42	15	0.4491

**Table 2H:** The influence of age on efficacy in the BSP group

age	N	Excellence	Effective	Invalid	P
18-30	26	8	11	7	0.4285
31-40	35	11	17	7	0.4494
41-50	55	18	28	9	0.4652
51-60	56	17	30	9	0.4541
61-70	28	8	17	3	0.5332

**Table 2I:** The influence of etiology on efficacy in the BSP group

Etiology	N	Excellence	Effective	Invalid	p
Trauma	68	21	35	12	0.4527
hormone	63	20	32	11	0.4575
Alcohol	23	7	9	7	0.4178
unknown	46	14	27	5	0.4677

**Table 2J:** X-ray improvement of different groups

group		Pre-treatment			post-treatment	
	**n**	**X±S**	**P**	**n**	**X±S**	**P**
BSP	100	3.13±1.06		100	2.81±1.05	
>0.05			<0.05			
Control	100	3.02±1.37		100	3.18±1.08	

I, II stage: 1 point; III stage: 2 point; IV stage: 3 point; V, VI stage: 4 point.

**Table 2K:** MRI improvement of different groups

Group		pretherapy			post-treatment	
	n	X±S	P	n	X±S	P
BSP	30	3.13±1.07		30	2.80±0.93	
>0.05			<0.01			
Control	30	3.14±0.98		30	3.07±0.88	

I, II stage: 1 point; III stage: 2 point; IV stage: 3 point; V, VI stage: 4 point.

## DISCUSSION

ONFH is a painful and debilitating condition, which affects both middle-aged and young adults [3, 22–24]. The pathogenesis and etiology of ONFH are complex and no single factor can fully explain how the blood supply to the femoral head is damaged, culminating in the death of hematopoietic cells, bone cells, and adipocytes [25–27]. As the blood vessels of the femoral head are minute with a large distribution angle and slow blood flow, they are susceptible to insults such as trauma and hormone-induced intravascular changes [28–30]. Although a number of studies have attempted to resolve the mechanism by which these insults trigger ONFH, effective medical treatment for this condition is still lacking [31, 32]. In traditional Chinese medicine theory, ONFH is considered as a type of bone erosion and bone atrophy, and the pathogenesis of which is Qi stagnation and blood stasis, meridians stasis, and marrow death. We according to the principles of TCM treatment of ONFH blood circulation, activate collaterals and blood bone. In this study, we used HPLC fingerprints to measure 10 samples of BSP and found 11 peaks. The major components of this pill are pberetima, pearl, angelica, and pseudo-ginseng. Moreover, the peaks of this pill did not change after 60 months of storage. We then established a rat model of ONFH induced by prednisone; ONFH occurred in 90% of prednisone-treated rats, and treatment with BSP caused 70% of rats to recover from ONFH. TBV, MTPT, CBV, and MOs W significantly decreased and the percentage of Nocy significantly increased. However, TBV, MTPT, CBV and MOs W significantly increased and the percentage of Nocy significantly decreased after treatment with BSP. Prednisone treatment significantly increased the number of cells with fat particles compared with those in control rats. In addition, the number of cells with fat particles significantly decreased after treatment with BSP. After prednisone treatment, the capillary vessels of the femoral head decreased and the arcuate cup of the capillary vessels disappeared; this was reversed by BSP treatment. Serum TRAP significantly increased and serum ALP decreased in ONFH rats compared with the values in control rats. These parameters were significantly improved by BSP treatment.

With the aim of translating the results of our animal study into clinical benefits, we used a double-blind, randomized trial to assess the potential therapeutic effect of BSP in 300 ONFH patients and found that the positive response rate was over 82%. Treatment with BSP was also accompanied by improved hip joint function, claudication, and imaging results as compared with the results reported for the control group. The response to BSP was independent of age, sex, and etiological subtypes (hormone, trauma, and alcoholic intemperance). Routine functional tests on different vital organs also demonstrated that BSP was safe. These data support the use of BSP as a therapy for ONFH in different patient subgroups. In addition, histological sections and MRI showed that the bone and cartilage cells of the femoral head before treatment had been damaged and lost. The damage to the femoral head was restored after BSP treatment. These results suggest that BSP can promote bone and cartilage repair. Experiments and Figure 2 show that BSP can increase the number of blood vessels and bone mineral density. Figure 3 and 4 show that ONFH repair is gradual from peripheral to center. This suggests that BSP may be restored by necrosis of the femoral head blood supply, activate and promote the surrounding normal bone repair and related factors (such as osteogenic cell— Stem Cell and osteoblast osteogenic function, etc.) to accelerate the proliferation and differentiation, crawling, substitution, bone tissue reconstruction.

However, the limitations of this study should not be ignored. First, the compounds that mediate the therapeutic effect of BSP in ONFH are still unclear. Similar to other traditional Chinese herbal mixtures, it is very difficult to identify the active ingredients of BSP given the potential synergistic actions among the different constituents. This leads directly to the second limitation: the molecular mechanism by which BSP improves ONFH is still uncertain. In conclusion, we have demonstrated that BSP is a safe and effective treatment for ONFH in both animals and humans, and acts partly by improving bone growth, promoting bone density, and restoring blood circulation to the femoral head. Bone necrosis disease is difficult to treat, and BSP may be an option to repair the damage. BSP could also be used to treat other types of orthopedic diseases.

## MATERIALS AND METHODS

### Ethics approval

The clinical part of this study was approved by the Research Ethics Committee of the Peking Union Medical College Hospital and Beijing JianxingTraditional Chinese Medicine Hospital. Human samples were obtained with written informed consent from each patient. Patients were from Peking Union Medical College Hospital, Wangjing Hospital, the China Academy of Chinese Medical Sciences, Shuguang Hospital, Shanghai University of Traditional Herbal Mixture, Fujian Academy of Traditional Herbal Mixture, and Hubei Academy of Traditional Herbal Mixture. Animal use for the experiment was approved by the Peking Union Medical College Hospital.

### High-performance liquid chromatography

High-performance liquid chromatography (HPLC) fingerprints of BSP were obtained with a Hitachi L-2130 series HPLC system equipped with a binary solvent delivery pump, a manual sampler manager, a column compartment, and an ultraviolet detector. Data were analyzed with Hitachi L-2000 software. Methanol of HPLC grade and other chemicals of analytical grade were from Tianjing Kermel Chemical Factory (Tianjin, China). Purified water generated by a Milli-Q water purification system (Millipore, Bedford, MA, USA) was used.

### Rat model of ONFH and treatment regimen

Wistar rats in the disease model group were subcutaneously injected with prednisone (10 mg/kg) for 12 weeks on consecutive days, while rats in the healthy control group received an equal volume of normal saline. Half of the animals in the disease model group were then given BSP in normal saline (2 ml) daily for 12 weeks, whereas the other half of the diseasemodel control animals received normal saline.

### Tissue harvesting and processing

Whole blood (5 mL) was obtained from all rats under general anesthesia, and the serum was aliquoted for biochemistry analysis. Femoral heads were collected from rats that were euthanized with xylazine plus ketamine. The femoral specimens were fixed with 10% neutral formalin at room temperature for 24 hours, and subsequently decalcified with 10% EDTA-Tris solution at room temperature for 4 weeks with the decalcification fluid changed every 3 days. A femoral head was considered completely demineralized if it could be pierced with a pin. All samples were then dehydrated with a series of graded ethanol washes, cleared with xylene for 2 hours at room temperature, and then embedded in paraffin. Tissue blocks were sliced into 4μm parasagittal sections with a rotary microtome, which were then processed for hematoxylin and eosin staining and terminal deoxynucleotidyl transferase dUTP nick end labeling (TUNEL). All slides were sealed with neutral resin and imaged with an inverted phase-contrast microscope equipped with a camera system.

### Alkaline phosphatase staining and activity assay

Alkaline phosphatase (ALP) staining was performed using a commercial kit (Millipore, Boston, USA) following the manufacturer’s instructions. Images were captured using a Leica microscope, and the ALP activity was determined using an ALP activity assay kit (Sigma) following the manufacturer’s instructions.

### Statistical analysis

All data are shown as mean ± standard deviation (SD). Student’s *t*test and one-way analysis of Variance (ANOVA) were performed to determine statistical significance using the SPSS 17.0 software (SPSS Inc., Chicago, IL, USA). A value of *P*< 0.05 was considered statistically significant.

## CONCLUSION

BSP, a natural medicine, can be used for the treatment of osteonecrosis including femoral head necrosis. BSP can inhibit and reverse, promote the repair of bone and cartilage diseases, thereby reducing the number of people in the labor force, increase every year for the community.
